# Ethnomedicinal Information on Plants Used for the Treatment of Bone Fractures, Wounds, and Sprains in the Northern Region of the Republic of Benin

**DOI:** 10.1155/2022/8619330

**Published:** 2022-12-21

**Authors:** Nonvignon Murielle Codo Toafode, Emelia Oppong Bekoe, Zacharie Vissiennon, Virgile Ahyi, Cica Vissiennon, Karin Fester

**Affiliations:** ^1^IRGIB-Africa University, Inter-Regional University of Industrial Engineering Biotechnologies and Applied Sciences, Cotonou, 07 BP 231, Benin; ^2^Leipzig University, Institute of Medical Physics and Biophysics, Leipzig 04107, Germany; ^3^University of Ghana, School of Pharmacy, Department of Pharmacognosy and Herbal Medicine, Legon, LG 43, Ghana; ^4^Repha GmbH Biologische Arzneimittel, Langenhagen 30855, Germany; ^5^University of Applied Sciences Zittau/Görlitz, Faculty of Natural and Environmental Sciences, Zittau 02763, Germany

## Abstract

Medicinal plants are frequently used in African countries due to their importance in the treatment of various conditions. In the northern Republic of Benin, traditional healers are recognized as specialists in the treatment of fractures, wounds, and sprains. The present study was conducted to document the practices (diagnosis and materials) and traditional knowledge accumulated by healers in this region on their area of specialty. In addition, literature-based research was performed to support the usage of the most cited plants. Sixty traditional healers identified as “reference persons” from Atakora and Donga departments in the northern Republic of Benin, who specialized in the treatment of fractures, wounds, and sprains, were interviewed in their communities through a semi-structured questionnaire. Information about the practice, age of the healers, medicinal plants used in this treatment, methods of preparation, and administration were collected. Samples of the plant species were also collected, identified, and stored in the national herbarium at the University of Abomey-Calavi, the Republic of Benin. The study enabled the identification of thirty-four (34) species belonging to twenty-three (23) families. *Ochna rhizomatosa* and *Ochna schweinfurthiana* (21%) were the most quoted plants among the species, followed by *Chasmanthera dependens* (12.1%), *Piliostigma thonningii* (11.3%), and *Combretum sericeum* (8.1%). These plants were reported to strengthen bones, reduce inflammation, relieve pain, and promote healing in the northern part of the Republic of Benin. Besides their ability to treat fractures, wounds, and sprains, they are also used for multiple purposes in the West African subregions. According to the available literature, some of the plants will need to be investigated for their phytoconstituents and pharmacological activity to validate their ethnobotanical uses. These results confirm the need for documenting traditional knowledge since it represents an opportunity for exploring plant species with potentially good pharmacological effects, which have been barely investigated. Plants identified may constitute a significant source of bioactive compounds in the treatment of various ailments such as skin inflammation and musculoskeletal disorders. They can be further explored to justify their use in traditional Beninese medicine.

## 1. Introduction

In sub-Saharan African countries, herbal medicine practice constitutes an important source of medicinal compounds essential in the health care system of the local population [1, 2]. Although modern medicine has made significant progress, the local population remains committed to the use of folk medicine. The reliance of the rural community on herbal medicine is still high in countries like the Republic of Benin [3]. Traditional medicine is based on knowledge, beliefs, and practices developed, preserved, and shared over generations [4]. Economic, social, and cultural factors have concurred in the preservation of these practices [5]. In Beninese, Ethiopian, and Indian traditional cultures, herbal medicine is used in the treatment of bone fractures [6–8], suggesting that phytochemicals may be promoted as a candidate therapy. They will contribute to shortening the healing period and enhancing the healing quality. A fracture can be described as a partial or entire separation in the continuity of the bone. For its repair, four overlapping stages such as hematoma formation, inflammation, repair, and remodeling are included [9]. In the case of an open fracture, the skin is injured, and this may lead to a microbial infection [10]. The practice of using herbal medicine to treat bone fractures may have been preserved due to different conditions including restricted access of the population to modern medicine and the unavailability of modern health facilities [11]. Therefore, rural dwellers in the Republic of Benin are still constrained to refer to traditional medicines for their common daily ailments. Thus, it is expected that substantial knowledge of the use of plants to treat bone fractures has been gathered in areas where plant use is widespread [12]. In the northern region of the Republic of Benin, traditional healers are known to be specialized in the healing of bone fractures, wounds, and sprains [13]. They are the main stakeholders in the transmission of the practice. The use of plants in the management of bone fractures, wounds, and sprains in the north area of the Republic of Benin is barely documented [13], which justifies the interest in collecting data of this knowledge through an ethnomedicinal study. Documenting this indigenous knowledge is crucial to the conservation and utilization of biological resources and the identification of bioactive compounds with therapeutic relevance [7]. The aim of the present study was to collect and document knowledge regarding the medicinal plants used by the local traditional healers in the treatment of bone fractures, wounds, and sprains; their usage, methods of preparation, and administration in the northern departments of the Republic of Benin (Atakora and Donga). In addition, a literature-based study was performed to provide an overview of the bioactive compounds and pharmacological activities of the most cited species, which support their usage in folk medicine as well as the ethnobotanical uses and phytoconstituents of identified species.

## 2. Materials and Methods

### 2.1. Study Area

**Figure 1:**
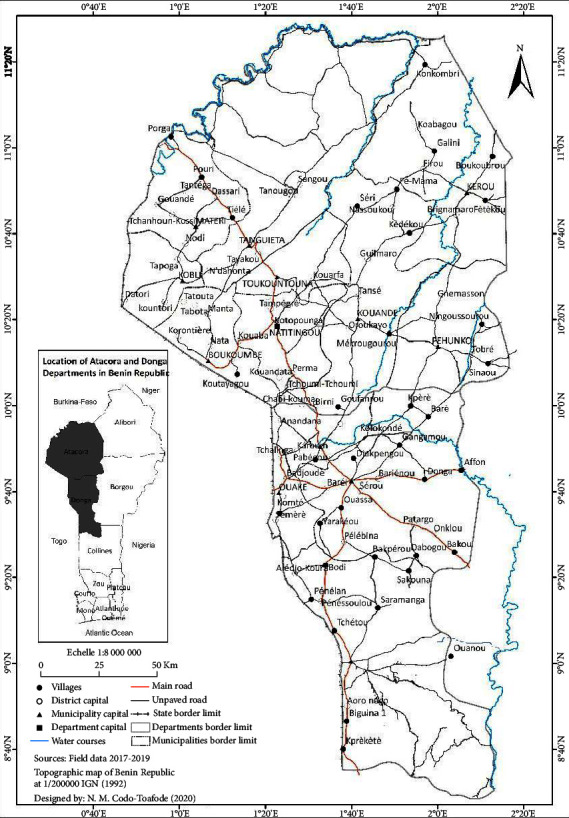
Map of Atakora and Donga departments.

The ethnomedicinal survey was performed in the Atakora and Donga departments in the northern region of the Republic of Benin (see Figure 1), and geographical locations of the healers were recorded with a GPS eTrex® Touch 25. Atakora is the most mountainous region of the Republic of Benin with 772,262 inhabitants, i.e., 5.4% of the Beninese population according to the fourth General Census of Population and Housing (RGPH4). Its coordinates are 10°45′0″*N*/1°40′0″*E*. Donga is bordering the Republic of Togo with 9°41′59.99″*N*/1°39′59.99″*E* as coordinates and constitutes 543,130 inhabitants of the Beninese population.

### 2.2. Ethnomedicinal Survey and Selection of Participants

The ethnomedicinal survey was conducted between February 2017 and January 2019 in agreement with the executive committee of the Federation of National Associations of Traditional Medicine Actors of Benin (FANAMETRAB), Republic of Benin. This study was authorized by the Research Ethics Committee of the Interregional University of Industrial Engineering, Biotechnology, and Applied Sciences under the number B001331455. As this work focuses on bone fractures, wounds, and sprains, traditional healers who specialize in the treatment of bone fractures and associated complications and are willing to share their practical knowledge were considered. Selection of the healers was based on the existing register of herbalists specialized in this area of treatment by the representatives of healers' association. The local population was of great help in locating them, especially in remote and hard-to-reach areas. Healers, who had specialized in the treatment of fractures, wounds, and sprains, were interviewed in almost every community. Participants were visited at their homes or workplaces and informed about the questionnaires. Informed consent of healers was obtained before administering the questionnaire. This process allowed the healers to feel valued and comfortable in their working environment. It also enabled us to collect relevant information about materials used for the treatment of patients. The questionnaire was designed in French and conducted either in French or the local language with the help of a translator or resident able to speak the local language. A pretesting of the semi-structured oral questionnaire with two healers in Natitingou was performed, and the questionnaire readjusted for an efficient collection of data. Sixty (60) healers specialized in the treatment of fractures, and associated complications have been interviewed. The full survey is available in the supplement. For dedicating time to the study and in respect of their tradition, a token was given to each healer. After the questionnaires and interviews were completed, healers were asked to provide samples of the plant materials mentioned. Samples were collected, labelled, and kept as voucher specimens for identification. Voucher specimens (see Table 1) of all plants were identified and deposited at the National Herbarium of the University of Abomey-Calavi, where each was given a defined ID-number.

### 2.3. Data Collection and Analysis

Questionnaires and interviews were used to collect biographical data of the respondents and details on their knowledge of the disease conditions (fractures, wounds, and sprains), years of experience, learning of practice, method of diagnosis, recipe, storage, and therapeutic usage of plants and plant parts used, their vernacular names, and the duration of treatment. Medicinal plants were rated based on how often the informants cited a particular plant. Data obtained from the ethnobotanical survey of medicinal plants used in the treatment of fractures, wounds, and sprains were recorded in Microsoft Excel spreadsheet software version 2016 and analyzed using descriptive statistical tools to determine frequency of citation (FC), informant consensus factor (*F*_ic_), percentages, and frequencies of quantitative data.

#### 2.3.1. Frequency of Citation

The frequency of citation (FC) of a species corresponds to the ratio between the number of respondents (*n*) who cited the species and the total number of respondents (*N*) and is calculated as follows [14]:

(1) $$\begin{array}{c} FC=\left(\frac{n}{N}\right)\;x\;100\text{.} \end{array}$$

#### 2.3.2. Informant consensus Factor

The informant consensus factor (*F*_ic_) was used to assess the homogeneity among informants concerning the relevance of plants used for the treatment of wounds, fractures, and sprains. It is calculated as the number of used citations in each category (*N*_ur_) minus the number of species used (*N*_*t*_), divided by the number of used citations in each category minus one [15]: *F*_ic_=*N*_ur_ − *Nt*/*N*_ur_ − 1.

The informant consensus factor (*F*_ic_) varies from 0 to 1. Values are low (close to 0) if plants are selected randomly or if no exchange of information about plants has occurred between informants. It approaches one (1) when there is a well-defined selection criterion in the community and/or if information is exchanged between informants.

### 2.4. Literature Validation of the Data

Plant species identified were confirmed using the Plant List (https://www.theplantlist.org/) for the accepted scientific names. Considering the probability of having the most cited plants according to our study reported in the scientific literature for treatment of fractures, wounds, and sprains, a cross-referencing was performed by entering their botanical names in combination with the search term “fracture, wound, or sprain” into the PubMed, Google Scholar, and ScienceDirect database. Databases were also used to look for available published data regarding the ethnobotanical uses and phytoconstituents of the plant species mentioned by the healers and reported in Table 2.

## 3. Results and Discussion

### 3.1. Ethnopharmacological Survey

#### 3.1.1. Geographical Distribution of Respondents

**Figure 2:**
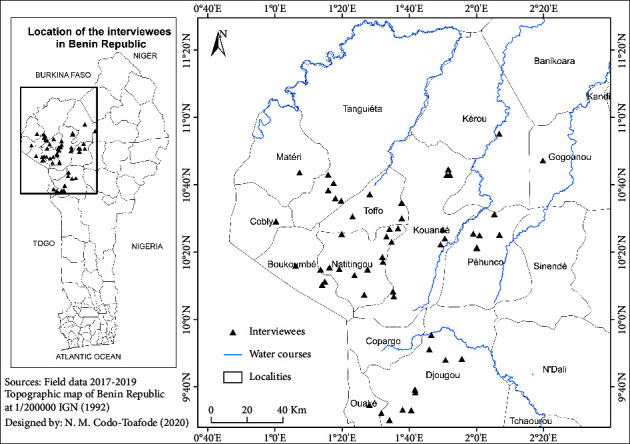
Geographical description of the study area. Map of Atacora and Donga departments, with triangles representing the coordinates of interviewed healers.

The geographical coordinates of each healer were recorded to have an insight into the geographical distribution in these departments (see Figure 2). Participants were well distributed in most of the communities, thus depicting the widespread dissemination of traditional medicine practiced by healers in rural areas.

#### 3.1.2. Demographic Characteristics of Surveyed Traditional Healers

The interview enabled the collection of socio-demographic information concerning the age, gender, educational level, ethnic group, and religion of the respondents who specialized in the treatment of fractures, sprains, and wounds. Healers were in the 21–90 age range, with 35% between (31–50) years and (51–70) years and 25% between (71–90) years. In addition, only 3% of healers were represented in the 21–30 age range. The gender distribution indicated that 90% of the practitioners were male. The socio-demographic characteristics of the traditional healers are presented in Table 1. The age distribution demonstrates that elders were more involved in this practice than young people, which was also revealed in studies carried out in Togo and Ethiopia [96, 97]. According to the results, women in this practice were in the minority, which confirms the findings of Upadhya et al. [8]. The number of patients visiting the healers depended on the season. During the rainy season, the poor and slippery conditions of the roads increased the number of accidents related to motorcycle use in the northern area of the Republic of Benin [98, 99]. According to the results, the level of education is such that 27% of the healers are educated, out of which 22% have attended primary school, 5% secondary school, and 73% have no education. The level of education of the healers in these areas is comparable to healers in the northern region of Ghana and the Republic of Benin [100, 101]. Healers belong to various ethnic groups like Waama, Ditammari, Bariba, Yiende, and Natimba in Atakora and Dindi, Yôme, Ani, Koura, Peulh, and Kotocoli in Donga. As far as religion is concerned, 23% of the healers were Christian, 25% Muslim, and 52% traditional, which justifies the fact that tradition is still strongly present in these departments, even if monotheistic religions are gradually setting in [3]. In addition to their healing practice, most healers engage in other livelihood. 88% are farmers, which is the predominant economic activity in these departments [102].

#### 3.1.3. Practical Knowledge of Healers in the Treatment of Bone Fractures, Wounds, and Sprains

The knowledge of treating fractures and its complications is, in most cases, passed on through generations. The skills obtained by practicing with a family member who has the experience or knowledge is complemented by one's own observations. The general process for treating fractures, wounds, and sprains can be described in three significant steps. Initially, an examination is made by the healer to identify the type of bone fracture or sprain. The next step of massage is performed by preparing an infusion or a decoction of plants that is applied to the injured part, followed by the next step of applying either the seed or the bark of the roots of plants in a powdered, incinerated, or chewed paste form. Regarding open fractures, all healers unanimously agreed that the wound must be healed before a treatment can be given. This report follows hospital practices where minimizing the risk of infection is a priority [10]. Sprains, open and closed fractures, and wounds can be treated by 97% of the healers while only 3% focus on the treatment of fractures. Thirty-one percent (31%) of the healers claim the ability to treat these conditions without the help of modern medicine. In contrast, the association with modern medicine such as radiography, the use of analgesics (e.g., paracetamol), sewing of the open wound before treating it, and collaboration with health workers is done by 69% of the healers. This, combined with modern medicine, is a proof that traditional medicine sometimes relies on modern medicine to ascertain a diagnosis [8]. The recovery time is variable, depending on the severity of the fracture. Two weeks are needed for a child to recover and 1–3 months for an adult. The application of the treatment is made once or twice a day, depending on the severity of the case. The materials needed in traditional medicine include mats, banana bark, and bandages, which are comparable to plaster casts used in modern medicine for immobilizing an injured part [103].

#### 3.1.4. Plant Species Cited and Their Applications for the Treatment of Fractures, Wounds, and Sprains

The ethnomedical study resulted in the identification of thirty-four (34) species representing twenty-three (23) families that are used for the treatment of fractures, wounds, and sprains (Table 3). For each plant species, a recipe has been collected that is valid for treating fractures, wounds, and sprains. Plants species are used with a technicality that varies depending on the type of condition to be treated (wounds, fractures, or sprains) with the same preparations. The most cited among the recorded species were *Ochna rhizomatosa* and *Ochna schweinfurthiana*, followed by *Chasmanthera dependens*, *Piliostigma thonningii,* and *Combretum sericeum.* They can be used either fresh or dried and are boiled, chewed, or grounded and burned to collect the powder before application. In addition, shea butter is used by 60% of the traditional healers as an excipient in the preparations, while 13.3% of them use either fats from beef, boa, and cow's cream or water, and 26.7% do not make use of excipients. The excipients are used to enable the easy application of the medicinal plant's preparation. The healers recognize the plant species using various criteria such as their morphology and habitat. The availability of plant materials can be influenced by different factors such as the season, the distance from harvesting, drought, and the extinction of plants due to bush fires [121].

#### 3.1.5. Informant Consensus Factor and Frequency of Citation

The informant consensus factor indicates a well-documented knowledge of medicinal plants by the respondents. The informant consensus factor (*F*_ic_) in this study is relatively high (0.79). This indicates the high degree of consensus among the healers on the plants used in the treatment of fractures, wounds, and sprains in the northern region of the Republic of Benin. *Ochna rhizomatosa* and *Ochna schweinfurthiana* turned out to have the highest frequency of citation (21%) followed by *Chasmanthera dependens* (12.1%), *Piliostigma thonningii* (11.3%), and *Combretum sericeum* (8.1%) in our study as shown in Table 3. The high frequency of citation of these plants demonstrates their frequent use by the healers and ranks them as essential plants in the treatment of fractures, wounds, and sprains.

### 3.2. Literature Review on Traditional Uses and Biological Activities of Cited Plant Species

A literature review was performed on the species with high frequencies of citation. Moreover, information on medicinal uses and chemical compounds of all the species identified is provided in Table 2. During interviews and collection of plant samples, it was observed that healers were using *Ochna rhizomatosa and Ochna schweinfurthiana* which were the most cited species for the treatment of bone fractures, wounds, and sprains. They constitute two different species but can be used alternatively in northern traditional Beninese medicine. They are called male (*Ochna rhizomatosa*) and female (*Ochna schweinfurthiana*) and are differentiated morphologically by the size of their leaves by the traditional healers. They describe them as having almost the same effect. They belong to the genus *Ochna*, which comprises ca. 86 species and contains a variety of flavonoids [122, 123].

The roots and stem bark of *Ochna rhizomatosa* have been identified as a plant used for the massage of the ribs in the form of a decoction in Ghana and Cameroon for the treatment of wounds, which strengthens its ethnopharmacological use in the Republic of Benin in the treatment of bone fractures, wounds, and sprains [100]. Egwu's [114] phytochemical study also shows the presence of a triflavonoid ester and biflavone derivatives, which were reported for the first time in *Ochna rhizomatosa* leaves. A recent study allowed for the identification of three biflavones ((R)-rhizomatobiflavonoid A-C) along with gerontoisoflavone A, schweinfurthianone A and B, and calodenine B and investigated their inhibitory effect against HIV and malaria [73]. Regarding *Ochna schweinfurthiana*, it is used in traditional medicine to treat pain, inflammation, skin infection, and arthritis [76, 124]. During the evaluation of the antimalarial potential of *Ochna schweinfurthiana* roots, several biflavones were identified, namely, calodenone, calodenine B, lophirone A, gerontoisoflavone A, and 4‴-methoxylophirone A, 4,4',4‴-trimethoxylophirone A [123]. A trimethoxy derivative of lophirone A has shown a potential antimicrobial effect [125]. In addition, other biflavones such as cupressuflavone and robustaflavone, together with epicatechin and 3-*β*-*O*-D-glucopyranosyl-*β*-stigmasterol compounds, were isolated from the ethyl acetate extract of the stem bark of the plant [77]. Recently, hemerocallone, amentoflavone, agathisflavone, lithospermoside, 6,7-dimethoxy-3′-4′-dimethoxyisoflavone, and *β*-D-fructofuranosyl-*α*-D-glucopyranoside were isolated from the acqueous extract of the bark of *Ochna schweinfurthiana* [124].

*Chasmanthera dependens* cited 15 times, has been reported for the management of fractures and wounds in Nigeria [30]. The species is known for its content of quaternary and tertiary phenolic alkaloid compounds [31]. It was shown to possess analgesic, anti-inflammatory, and antimicrobial properties [30, 126].

*Piliostigma thonningii* from the family Leguminosae, mentioned 14 times among healers constitutes an important species used in African traditional medicine. This plant is rich in flavonoids such as quercetin derivatives and C-methyl flavanols [84, 127]. In addition, a kaurane diterpenoid was isolated [128]. An in-depth investigation of *Piliostigma thonningii* resulted in the isolation of two compounds, methyl-ent-3-*β*-hydroxylabd-8(17)-en-15-oate and 2*β*-methoxyclovan-9*α*-ol along with 14 other known compounds, e.g., flavonoids compounds (quercetin-3-*O*-rhamnoside) and vitamins [85]. The plant has shown antibacterial and anti-inflammatory activities [127, 129], as well as a hepatoprotective effect [130].

*Combretum sericeum* from the family Combretaceae, and 10 times cited has been barely investigated. Members of the genus *Combretum* have shown interesting properties and are widely used as medicinal plants [131]. *C. sericeum* is described as having several ethnopharmacological effects, e.g., against diarrhoea and gastrointestinal disorders [36, 37], as well as antiplasmodial [132] and antimicrobial properties [133]. Despite the numerous features of this plant, only a few phytochemical studies have been performed. Tannins, terpenoids, saponins, various flavonoids, and anthraquinones were detected in the plant [134]. As seen from the literature discussed here, all the plants contain interesting bioactive components such as flavonoids and alkaloids. During bone fractures, wounds, and sprains, inflammation plays a significant role. The richness of these plants in various compounds may contribute to overcoming the inflammation process [135, 136]. This study demonstrates that medicinal plants used by traditional healers to treat fractures, wounds, and sprains may be potential candidates for pharmacological research focused on various conditions such as inflammatory skin and musculoskeletal conditions. In addition, the literature review on the ethnobotanical uses and phytoconstituents of the identified plant species revealed that *Rourea coccinea, Ipomoea pyrophila, Combretum sericeum,* and *Apodostigma pallens* require in-depth phytochemical studies. Finally, these investigations may lead to the development of reliable formulations of herbal medicines with proven clinical efficacy and confirmed safety.

## 4. Conclusion

The north of the Republic of Benin constitutes an area where the knowledge for treating bone fractures, wounds, and sprains is advanced. This ethnomedicinal study resulted in the documentation of the traditional medicine practice and provided an overview of plant species, their medicinal use, and mode of application in the treatment of bone fractures, wounds, and sprains. The recorded plants are administered as a decoction, infusion, or powder to strengthen bones, reduce inflammation, relieve pain, and promote healing. Based on these results, pharmacological and clinical assessments of these natural remedies can be conducted to rationalize their ethnomedicinal use and enhance the promotion of these plants.

## Figures and Tables

**Table 1:** Sociodemographic characteristics of traditional healers (*n* = 60) in the northern Republic of Benin.

Characteristics	Frequency of respondents	% number of respondents
*Gender*		
Male	54	90
Female	6	10

*Age-groups*		
21–30	3	5
31–50	21	35
51–70	21	35
71–90	15	25

*Educational level*		
No formal education	44	73
Primary school	13	22
Secondary school	3	5

*Ethnic group*		
Ditammari	15	25
Bariba	14	23.3
Waama	10	16.7
Yiende	5	8.3
Natimba	4	6.7
Koura	4	6.7
Dindi	2	3.3
Yôme	2	3.3
Peuhl	2	3.3
Ani	1	1.7
Kotocoli	1	1.7

*Religion*		
Traditionalist	31	52
Christian	14	23
Muslim	15	25

*Years of experience*		
5–10	12	20
11–20	21	35
21–30	13	21.6
31–40	7	11.7
>40	7	11.7

**Table 2:** Ethnobotanical uses and constituents of commonly used wound healing plants in the northern region of the Republic of Benin.

Botanical names	Ethnobotanical uses	Major phytoconstituents
*Adansonia digitata* L.	It is used as food (leaves and fruit), laxative, shelter (the tree), and in the treatment of diabetes, diarrhoea, trypanosomal diseases, and wounds [16, 17]	3,3′,4′-Trihydroxyflavan-4-one-7-*O*-*α*-L-rhamnopyranoside, quercetin-7-*O*-*β*-D-xylopyranoside, 3,7 dihydroxyl-flavan- 4-one-5-O-*β*-D-galacto pyranosyl (1 → 4)-*β*-D-glucopyranoside, campesterol, stigmasterol, cholesterol, isofucosterol, ß-sitosterol, and tocopherol [18]
*Aframomum melegueta* (Roskoe) K. Schum	Spice, flavoring agent, treatment of hemorrhoids, infections, abdominal pain, leprosy, measles, inflammatory disorders, and intestinal problems. It is used as purgative, galactogogue, and hemostatic agent [19]	Vanilloids, 6-paradol, 6-gingerol, monoterpene hydrocarbons and sesquiterpenes hydrocarbons [20], benzaldehyde-3-hydroxy-4-methoxy, butan-2-one-4-(3-hydroxy-2-methoxyphenyl), flavonoids (quercetin, kaempferol, and its derivatives), triterpenoids, labdane diterpenoids, zerumin A, labdane diterpenes G3 and G5, (E)-labda-8(17), 12-diene-15, and 16-dial saponins [21]
*Annona Senegalensis* Pers	The fruit is used for the treatment of marasmus and kwashiorkor. Treatment of diarrhoea, helminthiasis, microbial infections, snake bites, toothache, malaria, anenia, arthritis, inflammatory disorders, wound, dermatosis, and sexual impotence [22, 23]	1,2 Benzenediol, butylated hydroxytoluene (BHT), phenol, 2,6-bis (1,1-dimethylethyl-4methyl), methylcarbamate, *n*-hexadecanoic acid, hexadecane, oleic acid, tetracosane, 9-octylheptadecane, heneicosane, 12-methyl-E, E-2, 13-octadecadien-1-ol, 13-hexyloxacyclotridec-10-en-2one, octadecanoic acid, 9, 17-octadecadienal, pentadecane, tetratriacontane and squalene [23], kaurenoic acid, and (−)-roemerine [24]
*Apodostigma pallens* (Planch. ex Oliv.) R.Wilczek	Treatment of gonorrhoea [25]	—
*Ceiba pentandra* (L.) Gaertn	Treatment of asthma, fever, gonorrhoea, diarrhoea, diabetes, headache, and use as aphrodisiac (barks). Diuretic and febrifuge properties (roots). The seed oil is used as fuel and lubricant [26, 27]	Ceibapentains A and B, linarin, 3,4-dihydroxybenzoic acid, cinchonains ia and ib, N-trans-caffeoyl-DOPA-methyl ester, monoterpene, and sesquiterpene hydrocarbons [28, 29]
*Chasmanthera dependens* Hochst	Treatment of venereal diseases, fractures, sprains, muscular pains (leaves) and is used as uterine stimulant [30]	Berberine [30], tertiary nonphenolic alkaloids (tetrahydropalmatine, liriodenine, lysicamine, oxoglaucine, glaucine, anonaine, nornuciferine, norglaucine, *O*,*O*-dimethylcorytuberine, and anomaine), govanine (= (−) -tetrahydropseudocolumbamine), coreximine, bisnorargemonine, pallidine, and quaternary alkaloids (jatrorrhizine, columbamine, pseudocolumbamine, magnoflorine, and palmatine) [31, 32]
*Combretum collinum* Fresen	Treatment of wounds, ulcers, rheumatism, microbial infections, gastrointestinal problems, cough, and bronchitis with the leaves [33]	Myricetin-3-*O*-rhamnoside, myricetin-3-*O*-glucoside stilbenoids (combretastatins), phenanthrenes, and mollic acid [33]
*Combretum glutinosum* Perr. Ex DC	Treatment of scrotal elephantiasis, dysentery, typhoid fever, eye sore and earache, fever, stomachache malaria, diarrhoea, ring worms, bronchitis, hypertension, and syphilis [34, 35]	2,3-(5′)-Hexahydroxydiphenoyl-D-glucose, punicalin, punicalagin, umuhengerin and combreglutinin, betulinic acid, *β*-sitosterol glucoside, 5-demethylsinensetin, (20S,24R)-ocotillone, lupeol, *β*-sitosterol, oleanolic acid, and corymbosin [35]
*Combretum sericeum* G.Don	Treatment of diarrhoea, gastrointestinal disorders, conjunctivitis, fever, pneumonia, malaria, and use as repellent [36, 37]	—
*Crossopteryx febrifuga* (Afzel. ex G.Don) Bents	Treatment of dysentery, diarrhoea and fever [38], pain, malaria, septic wounds, and respiratory infections [39]	Epimeric mixtures of 3*β*-urs-12,20(30)-diene-27,28-dioic acid and 18-epi-3*β*-urs-12,20(30)-diene-27,28-dioic acid, 3*β*-D-glucopyranosylurs-12,20(30)-diene-27,28-dioic acid and 18-epi-3*β*-D-glucopyranosylurs-12,20(30)-diene-27,28-dioic acid, triterpenoid (*β*-chinovin), vitexin and isovitexin xylosides, quercetin-3-rutinoside, and alkaloids (crossopterine) [40]
*Eleusine indica* (L.) Gaertn	Use as diuretic, anthelmintic, febrifuge, diaphoretic, and treatment of cough [41]	Flavonoids schaftoside, vitexin, isovitexin, and 6′-*O*-palmitoyl derivatives, *β*-sitosterol, *β*-sitosterol glucoside, and stigmasterol [42]
*Entada africana* Guill. & Perr	Treatment of abdominal pain, rheumatism, malaria, female infertility [43], wounds, hepatitis, bronchitis, syphilis, and bronchitis [44]	Apigenin, robinetin, gallic acid, ethyl gallate, catechin, 5,7-dihydroxychromen-4-one, 3′,4′,7-trihydroxyflavone, naringenin-7-O-glucoside, myricetin-3-*O*-glucoside, dihydrokaempferol-7-*O*-glucoside, quercetin-3-*O*-glucoside, quercetin-3-*O*-(*β*-apiosyl-(1‴→2″)-*β*-glucoside), and aromadendrin [45]
*Feretia apodanthera* Delile	Treatment of epilepsy, infantile convulsions, anxiety, psychoses, pain, inflammation, and infective wounds [46, 47]	Iridoid glycosides (feretoside, gardenoside, geniposidic acid, apodanthoside, desacetylasperolosidic acid), quercetin, kaempferol, and myricetin [46, 47]
*Ficus ingens* (Miq.) Miq.	Treatment of epilepsy, leprosy, worm sore, injuries, hemorrhoids, diarrhoea and is used as a diuretic [48]	*β*-sitosterol, chryasophanol, 7-hydroxy-2,5 dimethylchromen-4-one, quercetin, aloe emodin glucoside, *β*-sitosterol glucoside, rutin, and patuletin-3′-*O*-methyl-3-*O*-rutinoside [49]
*Ficus thonningii* Blume	Treatment of diarrhoea, gonorrhoea, diabetes mellitus, wounds, bronchitis, urinary tract infections, stomach pains, gastritis, gastric ulcers, malaria, fever, and hepatitis [50]	Orientin, vitexin and isovitexin, thonningiol, thonningiisoflavone, *β*-sitosterol, *β*-sitosterol glucoside, gancaonin G, *β*-amyrin acetate, friedelin, lupeol hexanoate, lupeol acetate, alpinumisoflavone, wighteone, dehydroferreirine, *β*-isoluteone, taxifolin, lupiwitheone hydrate, *Rel (lR, 4S, 6R)-*p-menthane-3,6-diol, conrauiflavonol, aromadendrin, shuterin, luteone, and hydroxyalpinumisoflavone [51]
*Flueggea virosa,* (Roxb. ex Willd.) Royle	Treatment of fever, malaria, pain, diabetes, epilepsy, snakebites, rheumatism, arrhythmia, sterility, rashes, diarrhoea, pneumonia, sexual dysfunction, sexual impotence, cough, HIV-related illnesses, and venereal diseases [52]	Flueggenoids A–E, 13-methyl-ent-podocarpanes, and securinega alkaloids [53]
*Gardenia ternifolia* schumach. & Thonn	Treatment of malaria, stomachache, ulcers, malaria coughs, syphilis, arthritis, asthma, epilepsy, fever, pain. Use as purgative, laxative, astringent, and antisnake venom [54]	5,7-Trihydroxy-4′-methoxyflavone, 5,4′-dihydroxy-7-methoxyflavanone, 5,7-dihydroxy-3,4′-dimethoxyflavone, 3,5,3′-trihydroxy-7,4′-dimethoxyflavone, *β*-sitosterol and stigmasterol [54], and gardenifolins A–H [55]
*Gymnosporia senegalensis, L*. E. T. Loesener	Treatment of microbial infections, inflammation, tuberculosis, and respiratory diseases [56, 57]	Alkaloids (ephedrine, norephedrine, stachidrine, and wilforine) maytansinoids (maytanbutine, maytanprin, and maytanbutan), phenolic compounds, triterpenes, sterols (iguestrin, lupenone, *β*-sitosterol), and megastigmane [56, 57]
*Hannoa undulata* (Guill. & Perr.) Planch	Treatment of amoebic dysentery, malaria, anemia, and intestinal diseases [58]	Quassinoid (undulatone), polycyclic lactones (chaparrinone, klaineanone, and glaucarubolone), eniotorin, scopoletin, glaucarubinone, and 15-desacetylundulatone [58, 59]
*Hymenocardia acida,* Tul	Treatment of eye infection, sickle cell anemia, chest complaints, abdominal and menstrual pains, trypanosomiasis, coughs, hemorrhoids, stomachaches, microbial infections, asthma, and fractures [60]	Triterpenoids and sterols (friedelan-3-one, betulinic acid, stigmasterol, lupeol, *β*-sitosterol), fatty acid (oleic acid), hymenocardine, and hymenocardinol [61]
*Ipomoea pyrophila,* A. Cheval	—	—
*Jatropha curcas* L.	Treatment of eczema, skin diseases, rheumatic muscular pains, inflammation and is used as fuel [62]	Organic acids, cyclic triterpenes, stigmasterol, *β*-sitosterol, curcin, apigenin, vitexin, and isovitexin [63]
*Lannea microcarpa,* A. Rich	Treatment of fever, gastrointestinal problems, wounds, respiratory problems, ulcers, pain, malaria, musculoskeletal disorders, hemorrhoids, skin diseases and is used in ethnoveterinary medicine [64]	Epicatechin, myricetin glycosides, gallic acid, isovitexin, and cyanidin 3-*O*-*β*-D-galactopyranoside [64]
*Maerua angolensis,* DC.	Treatment of pain, cancer, fever, malaria, gastrointestinal problems, sores, and wounds [65]	Tannins, saponins, flavonoids, cardiac glycosides, and alkaloids [65]
*Newbouldia laevis* seem	Treatment of malaria, stomachache, toothache, wound dysentery, sexual transmitted disease (syphilis), migraine, and sexual impotency [66, 67]	Pyrazole alkaloids (withasomnine, 4′-hydroxywithasomnine, 4′-methoxywithasomnine, newbouldine, and 4′-hydroxynewbouldine), newbouldiosides D-F, 7-hydroxydehydroiso-*α*-lapachone, 5,7-dihydroxydehydroiso-*α*-lapachone and 3-hydroxy-5-methoxydehydroiso-*α*-lapachone, and 6-hydroxydehydroiso-*α*-lapachone [68–70]
*Ochna rhizomatosa (van tiegh.) Keay*	Treatment of intestinal helminthiasis, jaundice, malaria, wounds, and typhoid fever [71, 72]	(R)-Rhizomatobiflavonoid A-C, gerontoisoflavone A, schweinfurthianone A, shweinfurthianone B, and calodenine B [73]
*Ochna schweinfurthiana (van tiegh.) Keay*	Treatment of rubella, fungal skin infections, burns, stomachache, sclerosis, malaria, helminthiasis, typhoid fever, measles, and wounds [74–76]	Agathisflavone, cupressuflavone, sucrose, calodenone, calodenine B, robustaflavone, lophirone A, gerontoisoflavone A, amentoflavone, hemerocallone, 16*α*,17-dihydroxy-ent-kauran-19-oic acid and 3*β*-*O*-D-glucopyranosyl-*β*-sitosterol, 6,7-dimethoxy-3′,4′-dimethoxyisoflavone, epicatechin lithospermoside, and 3*β*-*O*-D-glucopyranosyl-*β*-stigmasterol [77]
*Ozoroa pulcherrima* (schweinf.) R. & A.	Treatment of asthenia, helminthiasis, conjunctivitis, chest pain, dystocia, hyperthermia, and conjunctivitis. It is used after childbirth to increase lactation [78–80]	Ozocardic A, 6-tridecyl anacardic acid, *β*-sitosterol, and ozoromide [78–80]
*Paullinia pinnata,* Linn	Treatment of snake bites, rabies, mental problems, blindness and eye troubles, paralysis, eczema, wounds, threatened abortion, malaria, ancylostomiasis, to expel placenta and gonorrhoea. [81]	3-Oxo-11*α*-hydroxyl-20-lupene, lupeol-3-isovanniloyl ester, 5*α*-portiferastane-3*β*,6*α*-diol and 2-(4-hydroxyl-3,5-dimethoxyl phenyl)-3-hydroxymethyl-2,3-dihydro-1,4,5-trioxaphenanthrene-6-one, lupeyl steryl ether [82]
*Piliostigma thonningii,* (schum.) Milne-Redh	Treatment of malaria, leprosy, wounds, ulcers, gingivitis, fever, cough, toothache, sore throat [83] Treatment of dysentery, diarrhoea, inflammation, skin diseases, and intestinal problems [84–86]	4-Hydroxybenzoic acid, piliostigmin, afzelin, quercitrin, 7 (9-desoxy-*α*-conidendrin, quercetin-3-*O*-rhamnoside, 2*β*-methoxyclovan-9*α*-ol, and methyl-*ent-3β*-hydroxylabd-8(17)-en-15-oate, (3*α*R,9R,9*α*s)-7-hydroxy-9(4-hydroxy-3-methoxyphenyl)-6-methoxy-3*α*,4,9,9*α*-tetrahydro-1H benzo[*f*] [2] benzo-furan-3-onenabellamide), 3,4-dihydroxybenzoic acid, (2E)-3-(4-hydroxy-3-methoxyphenyl) prop-2-enoic acid, and 8-(*β*-D-glucopyranosyl)-4′,5,7-trihyroxyflavone [84, 85]
*Rourea coccinea* (schumach. & Thonn.) Benth	Treatment of paralysis, Alzheimer's disease, snakebites, sexual asthenia, furuncles, malaria, joint pains, asthma, male and female infertility, and blennorrhoea [87]	—
*Sarcocephalus latifolius,* J. E. Smith et E.A. Bruce	Treatment of fever, toothache, dental cures, septic mouth, diarrhoea, and dysentery; used as chewing stick [88]	Indole alkaloids (augustine, nauclifine, augustidine, 21-*O*-methylstrictosamideaglycone, 19-*O*-ethylaugustoline, naucleidinal, 19-epinaucleidinal), *β*-sitosterol, tramadol, naucleamide, quinovic acid-3*β*-*O*-*β*-D-fucopyranoside, quinovic acid-3*β*-*O*-*β*-D-rhamnopyranoside, and scopoletin [88, 89]
*Vitellaria paradoxa* C.F.Gaertn. ssp. *paradoxa*	Treatment of cancer, diarrhoea, hemorrhoids, cough, tuberculosis, infectious diseases, headache hypertension, malaria and is used to relieve labor and delivery pains [90]	Triterpene acids of oleanane-type and glycosides (paradoxosides A-E, parkiosides A-C, tieghemelin A), flavonoids (catechin, gambiriin C, luteolin-7-glucoside, myricitrin, quercetin) [90]
*Vitex doniana* sweet	Treatment of diabetes, high blood pressure, ulcers, swellings, and oedema [91]	2,3-Acetonide-24- hydroxyecdysone, 21-hydroxyshidasterone, 11*β*-hydroxy-20-deoxyshidasterone, ajugasterone, 11*β*,24-hydroxyecdysone, ecdysteroids shidasteron, and 24-hydroxyecdysone [92]
*Xylopia aethiopica* (Dunal) A. Rich	Treatment of menstrual disorder, naso-pharyngeal infections, arthritis, rheumatism, diarrhoea, dysentery, cough, malaria, uterine fibroid, wounds, and stomach disorder [93, 94]	Diterpenes (15-oxo-(−)-trachyloban-19-oic acid, (−)-kaur-15-en-17-al-19-oic acid), and terpenes [95]

**Table 3:** Medicinal plants used for the treatment of bone fractures, wounds, and sprains.

Scientific names	Plant family	Local names^*a*^	Voucher N*°*	Fre-quen-cy^*b*^	Plant parts used^c^	State of plant parts	Modes of preparation	FC (%)	Literature reference^*b*^
*Adansonia digitata* L.	Malvaceae	^*B*^Sônou	YH 443/HNB	1	B	Fresh	Decoction of the bark of the trunk for the massage	0.8	[104]
*Aframomum malegueta* [Roskoe] K. Schum	Zingiberaceae	^*D*^Fètcharinanfè	YH 444/HNB	2	Se	Dried	Powder of the seeds applied topically	1.6	[22]
*Annona senegalensis* Pers	Annonaceae	^*B*^Batoko	YH 445/HNB	1	L	Fresh/dried	Infusion of the leaves for the massage	0.8	
*Apodostigma pallens* (Planch. ex Oliv.) R.Wilczek	Celastraceae	^*D*^Mukentetie	YH 446/HNB	2	L and R	Fresh/dried	Infusion of the leaves for the massage; chewing of roots applied topically	1.6	
*Ceiba pentandra* (L.) Gaertn.	Malvaceae	^B^Direbou billa, ^*w*^Kunkunfa	YH 448/HNB	2	Se and B	Fresh/dried	Powdered seeds in association with other plants; the bark is used as a mat	1.6	
*Chasmanthera dependens* Hochst	Menispermaceae	^*B*^Boborou, ^*P*^silpèrèya	YH 449/HNB	15	L and R	Fresh/dried	Infusion of the leaves for the massage; Grinded stem applied topically and sometimes mixed with shea butter	12.1	[30]
*Combretum collinum* Fresen	Combretaceae	^*B*^Gberukporo,^*D*^Tipèpèti	YH 450/HNB	2	L and R	Fresh/dried	Infusion of the leaves for the massage; bark of the roots in powder applied topically.	1.6	[33]
*Combretum glutinosum* Perr. Ex DC	Combretaceae	^Yi^Oudadaribou	YH 451/HNB	1	L and R	Fresh	Infusion of the leaves for the massage; incineration of the powdered bark of the roots applied topically	0.8	[105, 106]
*Combretum sericeum* G.Don	Combretaceae	^*W*^Cocopourka, ^*B*^soossi	YH 452/HNB	10	L and R	Fresh/dried	Infusion of the leaves for the massage; powdered bark of the roots applied topically.	8.1	
*Crossopteryx febrifuga* (Afzel. ex G.Don) Bents	Rubiaceae	^Yi^Otoupedou	YH 453/HNB	1	L and R	Fresh/dried	Infusion of the leaves for the massage; powdered bark of the root applied topically.	0.8	[39]
*Eleusine indica* (L.) Gaertn	Poaceae	^*W*^Yandé	YH 454/HNB	1	L	Fresh/dried	Infusion of the leaves + decoction of the bark of *C. pentandra* for the massage	0.8	
*Entada africana* Guill. & Perr	Fabaceae	^*B*^Wondorou	YH 455/HNB	1	L	Fresh/dried	Infusion of the leaves for the massage	0.8	
*Feretia apodanthera* Delile	Rubiaceae	^*N*^Diebaata	YH 456/HNB	1	L	Fresh	Infusion of the leaves for the massage	0.8	[107]
*Ficus ingens* (Miq.) Miq	Moraceae	^*B*^Dekuru sanni	YH 457/HNB	1	L and R	Fresh/dried	Infusion of the leaves for the massage; powdered bark of the roots applied topically.	0.8	[108]
*Ficus thonningii* Blume	Moraceae	^*K*^Kudoro	YH 458/HNB	1	L and R	Fresh	Infusion of the leaves of *F. thonningii* + *N. laevis* for the massage. Powdered bark of the roots of *N. laevis* + *F. thonningii* adventitious roots applied topically.	0.8	[109]
*Flueggea virosa,* (Roxb. ex Willd.) Royle	Phyllanthaceae	^Yi^Opanko, ^Yi^ipèènki, ^*N*^N'Dadaagui ^*N*^Yerikilangou ^*N*^N'Dadaaditchi	YH 459/HNB	5	L and R	Fresh/dried	Infusion of the leaves used orally; incineration of bark of roots applied topically	4.0	[52]
*Gardenia ternifolia* schumach. & Thonn	Rubiaceae	^Yi^Keyabouaka	YH 460/HNB	1	L and R	Fresh	Infusion of the leaves for massage; incineration of the bark of the roots mixed with the kernel oil butter applied topically	0.8	
*Gymnosporia senegalensis, L*. E. T. Loesener	Celastraceae	^*D*^Moukorou, ^*B*^Gberamonro-kou	YH 461/HNB	2	R	Fresh/dried	Powdered bark of the roots applied topically	1.6	
*Hannoa undulata* (Guill. & Perr.) Planch	Simaroubaceae	^*B*^Okoupopode	YH 462/HNB	1	L and R	Fresh/dried	Infusion of the leaves for the massage. Powdered bark of the roots applied topically	0.8	
*Hymenocardia acida,* Tul	Phyllanthaceae	^*B*^sinkakakou	YH 463/HNB	4	L and R	Fresh/dried	Infusion of the leaves for the massage. Powdered bark of the roots applied topically	3.2	
*Ipomoea pyrophila,* A. Cheval	Convolvulaceae	^*D*^Timonyati/^*D*^Tiwontèwonti	YH 464/HNB	2	L	Fresh/dried	Infusion of the leaves for the massage	1.6	
*Jatropha curcas* L.	Euphorbiaceae	^Yô^Kokofeku	YH 465/HNB	1	L	Fresh/dried	Infusion of the leaves for the massage	0.8	[110]
*Lannea microcarpa,* A. Rich	Anacardiaceae	^*B*^sinman, ^D^Mupèitèsinhou	YH 466/HNB	4	L and R	Fresh/dried	Infusion of the leaves for the massage; powdered bark of the roots applied topically.	3.2	[111]
*Maerua angolensis,* DC	Capparaceae	^Yi^Fetounanfè	YH 467/HNB	1	L and R	Fresh/dried	Infusion of the leaves for the massage; powdered bark of the roots applied topically.	0.8	
*Newbouldia laevis* seem	Bignoniaceae	^*K*^Abountou	YH 468/HNB	1	L and R	Fresh	Infusion of the leaves of *F. thonningii* + *N. laevis* for the massage.	0.8	[112, 113]
							Powdered bark of the roots of *N. laevis* + *F. thonningii* adventitious roots applied topically		
*Ochna schweinfurthiana/Ochna rhizomatosa* (van tiegh.) Keay	Ochnaceae	^*W*^Yinkpenoka, ^*D*^Mukentètié, ^*B*^Gounougokpes-sio	YH 469/HNB YH 470/HNB	26	L and R	Fresh/dried	Infusion of the leaves for the massage; powdered bark of the roots applied topically	21.0	[77, 114]
*Ozoroa pulcherrima* (schweinf.) R. & A.	Anacardiaceae	^*D*^Mukentétié	YH 471/HNB	2	L and R	Fresh/dried	Infusion of the leaves for the massage; powdered bark of the roots applied topically	1.6	
*Paullinia pinnata,* Linn	Sapindaceae	^*W*^Dikitinintibou	YH 472/HNB	2	L and R	Fresh/dried	Infusion of the leaves for the massage; powdered bark of the roots applied topically	1.6	[115–117]
*Piliostigma thonningii,* (schum.) Milne-Redh	Fabaceae	^*D*^Tilabaati, ^*B*^Bakourou, ^*N*^Nambaati	YH 473/HNB	14	L and R	Fresh/dried	Infusion of the leaves for the massage; powdered bark of the roots applied topically	11.3	[117]
*Rourea coccinea* (schumach. & Thonn.) Benth	Connaraceae	^*W*^Tchekidafa	YH 474/HNB	1	L	Fresh	Infusion of the leaves for the massage	0.8	[118]
*Sarcocephalus latifolius,* J. E. Smith et E.A. Bruce	Rubiaceae	^Yi^Oukôkômou, ^*W*^Comgonmou, ^*D*^Tikocoti	YH 475/HNB	4	L	Fresh/dried	Infusion of the leaves for the massage	3.2	[119]
*Vitellaria paradoxa* C.F.Gaertn. ssp. *paradoxa*	Sapotaceae	^*B*^somou	YH 447/HNB	2	L	Fresh/dried	Infusion of the leaves for the massage; mixing of the butter with powdered plants applied as an ointment	1.6	[120]
*Vitex doniana* sweet	Verbenaceae	^*N*^Hampou	YH 476/HNB	1	L	Fresh	Infusion of the leaves for the massage	0.8	
*Xylopia aethiopica* (Dunal) A. Rich	Annonaceae	^*W*^Nadofacha, ^p^ Kimadjè	YH 477/HNB	8	Se	Dried	Powdered seeds applied topically	6.5	

^*a*^Local name of the plant species: ^*B*^(Bariba), ^*D*^(Ditammari), ^*K*^(Koura), ^*N*^(Natimba), ^*P*^(Peuhl), ^*W*^(Waama), ^Yi^(Yiende), and ^Yo^(Yôme). ^*b*^Frequency indicates the number of times species were mentioned and recorded by the 60 healers. ^*c*^Plant indicates parts that were used: L (leaf), B (bark), R (root), S (stem), and Se (Seed).

## Data Availability

All data generated or analyzed during this study are included in this published article and its supplementary information files.

## Abbreviations

FANAMETRAB: Federation of National Associations of Traditional Medicine Actors of Benin
*C. sericeum*: *Combretum sericeum*
*N. laevis*: *Newbouldia laevis*
*F. thonningii*: *Ficus thonningii.*

## Supplementary Materials

Supplementary Materials Supplementary material file is entitled “Survey of the collection of data on plants used in the treatment of fractures, wounds, and sprains.”
