# Supplementary material for: Ethnomedicinal Information on Plants Used for the Treatment of Bone Fractures, Wounds, and Sprains in the Northern Region of the Republic of Benin
Source: Evid Based Complement Alternat Med. 2022 Dec 21;2022:8619330. doi: 10.1155/2022/8619330 (PMC9797300; doi:10.1155/2022/8619330)
Supplement: Supplementary Materials — Supplementary material file is entitled “Survey of the collection of data on plants used in the treatment of fractures, wounds, and sprains.” [file 8619330.f1.docx]

**Survey of the collection of data on plants used in the treatment of fractures, wounds and sprains**

**Survey N°**

Date :………………………….

Identity of the respondent

Name:………………………….. Localiy:………………… Socio-cultural group: ……………………

Age: ……………………………. Sex:……………………... Year of experience: …………………….

Level of education:………………………………….. Social status:……………………………… ...……………….b….…..:…………….……………………………....…………….,…………:………………………………………………….

1. Knowledge of the disease
2. What is a fracture / sprain?

………………………………………………………………………………………………………………………………………………………………………………………………………………………………………………………………………………………………………………………………………

1. What is a fracture / sprain in your language called?

……………………………………………………………………………………………………………………………………………………………………………………………………

1. When is a fracture/ sprain and what are the signs of a fracture/ sprain? ………………………………………………………………………………………………………………………………………………………………………………………………………………………………………………………………………………………………………………………………………
2. What types of fractures do you treat?

Open fractures Closed fractures

1. How many cases do you treat per week?

……………………………………………………………………………………………………………

1. Is the treatment done by :

- Plants only - Modern Medicine - Association

1. What are the treatment steps?

………………………………………………………………………………………………………………………………………………………………………………………………………………………………………………………………………………………………………………………………………

1. Knowledge of the plants used

1. Which plants do you use to treat fractures and how do you identify them?

…………………………………………………………………………………………………………………………………………………………………………………………………………………………………………………………………………………………………………………………………………………………………………………………………………

1. Which parts are used?

| Plants | Leaves | Bark | Roots | Stems | Fruits | Others |
| --- | --- | --- | --- | --- | --- | --- |
|  |  |  |  |  |  |  |
|  |  |  |  |  |  |  |
|  |  |  |  |  |  |  |
|  |  |  |  |  |  |  |
|  |  |  |  |  |  |  |
|  |  |  |  |  |  |  |
|  |  |  |  |  |  |  |

1. How do you call these plants in your language? (Brief description)

| Plants | Indigenous name | Scientific name (to be filled in after) |
| --- | --- | --- |
| 1 |  |  |
| 2 |  |  |
| 3 |  |  |
| 4 |  |  |
| 5 |  |  |
| 6 |  |  |

1. Which other pathologies do these plants treat?

| Plants | Pathologies / food use |
| --- | --- |
|  |  |
|  |  |
|  |  |

1. What is the dosage and duration of treatment (adult/youth)?

………………………………………………………………………………………………………………………………………………………………………………………………………………………………………………………………………………………………………………………………………

1. What are the method of harvesting and the state of the plant matter (Plant near or far)?

………………………………………………………………………………………………………………………………………………………………………………………………………………………………………………………………………………………………………

1. In which form is (are) used the plant(s) and what is the method of preparation (infusion, decoction, maceration, herbal tea, extract).

………………………………………………………………………………………………………………………………………………………………………………………………………………………………………………………………………………………………………………………………………

1. Does the use of the plant require the use of excipients?

……………………………………………………………………………………………………………………………………………………………………………………………………

1. Have any side effects been reported after the use of this (these) plant(s)?

…………………………………………………………………………………………………………………………………………………………………………………………………………………………………………………………………………………………………………………………………………………………………………………………………………

Signature of Researcher
